# Intratumor heterogeneity: A new perspective on colorectal cancer research

**DOI:** 10.1002/cam4.3323

**Published:** 2020-08-27

**Authors:** Zicheng Zheng, Tao Yu, Xinyu Zhao, Xin Gao, Yao Zhao, Gang Liu

**Affiliations:** ^1^ Department of General Surgery Tianjin Medical University General Hospital Tianjin China; ^2^ Tianjin General Surgery Institute Tianjin China; ^3^ Department of Oncology Tianjin Medical University General Hospital Tianjin China

**Keywords:** clonal evolution, colorectal cancer, intratumor heterogeneity, prognosis, review, sequence analysis

## Abstract

Colorectal cancers generally consist of multiple subclones. These subclones have their own unique characteristics, resulting in intratumor heterogeneity (ITH). As the discussion of ITH has advanced, a model describing the relationship of ITH to the tumor has gradually emerged. ITH can be divided into two types of intraprimary tumor heterogeneity and intraindividual tumor heterogeneity, the former for further understanding of tumor composition, and the latter for providing more information about evolutionary patterns. With the rapid development of new methods, such as next‐generation, polyguanine region sequencing, and Image detection, researchers may unravel the secrets underlying ITH. The higher the ITH of the tumor, the richer the interaction between the subclones maybe, or the greater the chance of the tumor getting more powerful subclones may be, thus increasing the malignant potential of the tumor. Existing evidence suggests that ITH may increase the ability of tumors to resist treatment and can be used as an independent influence on the prognosis of colorectal cancer. We reviewed 80 recent studies to give researchers a new perspective on colorectal cancer. There is still a limited amount of research in this area. Further study of the relationship between ITH and clinical endpoints may lead to the development of new treatment strategies.

## Graphical abstract

Intratumor heterogeneity may reveal the course of tumor development, progression, and metastasis, and may affect prognosis. The concept of intraprimary tumor heterogeneity and intraindividual tumor heterogeneity proposed by us may help to further enhance researchers' understanding of this field.

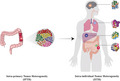

## 1 INTRODUCTION

Colorectal cancer (CRC) is the fourth most commonly occurring cancer among men. Globally, it is the third and fourth leading cause of cancer‐related deaths in men and women, respectively, and it represents one of the major diseases that threatens human health.^1^, ^2^ The uncontrollable proliferation and metastasis of cancer cells within major organs, such as the liver and lung, are the leading causes of death in CRC patients.^3^, ^4^ Surgical resection is generally used for CRC patients that do not present with metastasis, however, resection of the primary lesions is often insufficient.^5^, ^6^ Cancer recurrence in major organs following resection remains a significant cause for treatment failure. To improve clinical outcome in patients with recurrent CRC, oncologists rely on gene testing to select eligible drugs. During this process, they are often surprised to find that there are differences in mutated sites and biomarkers between tumors from the primary site and the matched metastasis.^7^ Over time, researchers have discovered that colorectal cancers are not homogeneous, but rather heterogeneous, consisting of many different cells or subclones of which different gene expression profiles among them.^8^ These differences within the tumor are referred to as intratumor heterogeneity (ITH). Heterogeneity endows tumors with multiple capabilities and biological characteristics, making them more prone to metastasis, recurrence, and drug resistance.^9^

**FIGURE 1:**
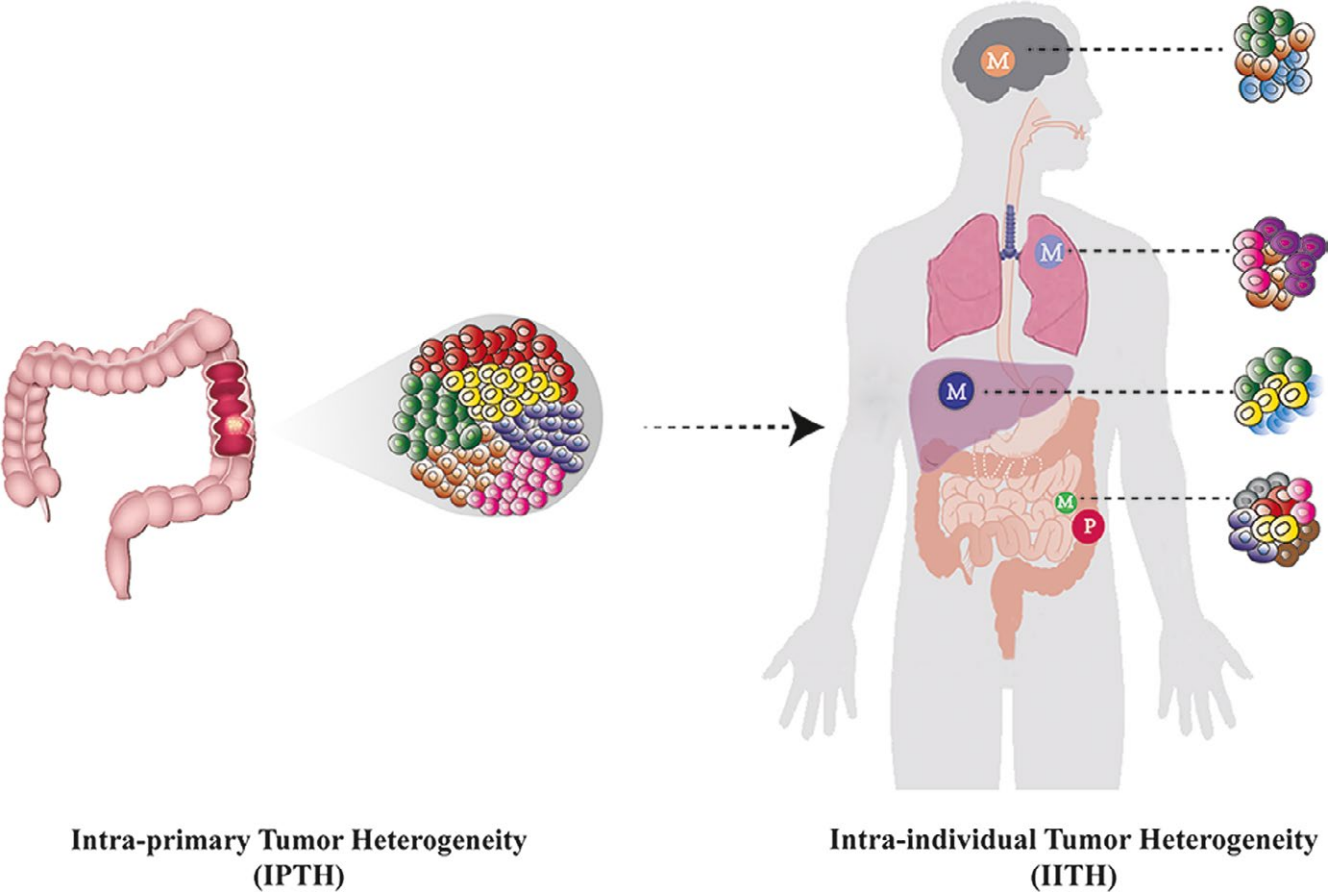
Intratumor heterogeneity (IPTH) and intraindividual heterogeneity (IITH) in colorectal cancer. (IPTH) This schematic image simulates a primary tumor in the descending colon composed of six distinct subclones (red, yellow, green, light brown, blue, and pink). Within the tumor, these subclones are clustered in different parts of the tumor. Each subclone grows in its own space and can be mixed at the boundary. The heterogeneity of primary tumors results from these subclones. (IITH) As a tumor progresses, different subclones may metastasize to perienteric lymph nodes and distant organs such as liver, lung, and brain. Perienteric lymph nodes were metastasized by subclones of four primary tumors (yellow, red, blue, and pink), with the birth of two private subclones (brown and gray, respectively). The liver was metastasized from subclones of two primary tumors (yellow and green, respectively) and a private subclone (sky blue) was born. Similarly, the lung has a subclone of two primary tumors (pink and light brown, respectively) and a private subclone (purple). There are two primary subclones of the brain (green and light brown) and a private subclone (sky blue). The schematic diagram indicates that all metastases are related to the primary tumor. The metastasis of lung and liver was similar to that of the perienteric lymph nodes, with the possibility of linear metastasis. Brain metastasis was different from perienteric lymph nodes, suggesting parallel metastasis. There are even replants from liver metastases. (P: primary site; M: metastatic site)

In the classic CRC development pathway, an adenoma undergoes a shift to become an adenocarcinoma. Successive mutations occur in the *APC*, *TGF‐beta*, *RAS,* and *TP53* genes during this process.^10^ As tumor cells divide, these mutations occur randomly, accumulate and are transmitted to their offspring, resulting in multiple subclones with different genotypes.^11^ Upon inspection, the primary tumor now consists of a collection of different subclones. At this stage, the heterogeneity in the primary tumor lesion may be referred to as intraprimary tumor heterogeneity (IPTH) (Figure 1). IPTH can provide us with a clearer understanding of the internal composition of tumors. Furthermore, cancer is so pernicious that it will never be confined to its original position. There are several existing modes of cancer metastasis including parallel progression, linear progression, dormancy mode, and tumor self‐seeding.^12^ However, ITH also occurs at the metastatic lesion regardless of the specific mode.^13^ The differences between the primary and matched metastatic lesions are known as intraindividual tumor heterogeneity (IITH) (Figure 1). IITH may act as a tracker to explore patterns and mechanisms of cancer metastasis. For the first time, the borderline between IPTH and IITH can be distinguished and this will provide a theoretical foundation for future research.

In recent years, the development of next‐generation sequencing (NGS) technology has facilitated the study of ITH. Many studies related to ITH have been published. In this review, we summarize the research progress made with respect to ITH in CRC. ITH may be the key to understanding the molecular basis for the evolution of CRC and other cancers.

## 2 ITH REFLECTS THE EVOLUTIONARY PATHWAY OF CRC

With respect to ITH, it is important to understand the evolutionary path of cancer. ITH results from branched evolution, which is the natural product of mutation.^14^ Mutations accompany tumor development during the growth of CRC and occur over the evolutionary course of a tumor. Because most mutations are neutral, they continue to spread as tumor cells proliferate. Over time, mutations accumulate and genotypes become abundant and diverse.^15^ Accumulation of new mutations eventually leads to qualitative transformation and promotes tumor evolution.^16^ Due to genetic drift, the variant allele frequency (VAF) may gradually increase in tumor cells.^17^ The emergence of selection caused by various factors further changes the proportion of mutant genotypes in CRC. Eventually new subclones will carry these mutations because of the proliferation of tumor cells.^18^ As a result, ITH emerges from CRC.

Although ITH appears to amplify the effect of mutations under selection, the timing of the specific mutation that leads to the creation of the new subclone is critical. It is possible that the biological characteristics of tumors are established early in the course of development, and tumor progression plays a role in subsequent amplification and selection.^19^ In the early stage of tumorigenesis, there are public mutations shared by all subclones and private mutations that occur during the process of replication. Private mutations produced in the early stage of tumorigenesis are trunk mutations, which gradually evolve into multiple subclones that are distributed to different regions of the tumor.^20^ Although new private mutations may occur during tumor cell proliferation, Korolev et al^21^ reported that the continual selective sweep that leads to the alteration of subclone structure is rare. Therefore, it is difficult for subsequent private mutations to produce new subclones. Some studies further support this idea. Patients with precancerous lesions accumulated more driver mutations and exhibited a higher VAF compared with advanced CRC patients.^22^ Multiple trunk APC mutations and heterogeneity of *KRAS* mutations were also found in early colorectal adenomas, suggesting that ITH is already present in such lesions.^23^ The early evolutionary progress of CRC is branched, while most mutations in advanced CRC are neutral. Time is a natural process that enables the expansion of the early selection effect.

In addition to coding DNA, there are other factors that can produce ITH. In the field of noncoding DNA, the impact of microsatellite instability (MSI) status to ITH is somewhat controversial. Studies have found that ITH in MSS is higher than MSI‐H, which may be due to the ITH of MSI‐H tumors reduced by tumor infiltrating lymphocytes and M1 macrophages.^24^, ^25^ While some researchers have discovered that CRC patients with MSI‐H have higher ITH, this may pertain to the increased mutation rate caused by mismatch repair gene defects.^26^ Chromosome instability (CIN) can also cause ITH through the gain or loss of large segments of the tumor genome, or by a change in the ploidy of chromosomes.^27^ ITH resulting from these phenomena may also provide more information with respect to the evolutionary pathway of CRC.

## 3 GENE SEQUENCING, IMAGING, AND CELL CULTURE ARE THREE "WINDOWS" TO EXPLORE ITH

The ITH of CRC is represented by subclones distributed in different spaces.^28^ In order to identify these subclones, the most common method is to obtain tumor VAF. This relies on NGS to detect DNA information or RNA sequencing to indirectly reflect genetic information to obtain single nucleotide variant (SNV), indel, or copy number variation (CNV) to calculate VAF.^29^ Cancer cell fraction (CCF) also needs to be taken into account to assess the reliability of VAF.^30^ After the subclones of each sample have been identified, it is common to construct phylogenetic trees from multiple samples using Bayesian methods in order to obtain more information about the evolutionary patterns of tumors.^31^ Once a specific value of bootstrapper (usually greater than 70) is set, each sample composed of multiple subclones can be recombined into a different trunk, and the number of branches of the evolutionary tree and the genetic distance between the samples can be determined.^32^ In addition, other algorithms for constructing the phylogenetic trees, such as Binary tree,^33^ are also emerging gradually. In recent years, single‐cell sequencing has provided a hopeful method for building evolutionary trees by providing cell‐level genetic information, which has been partially confirmed in some studies.^34^, ^35^ Some researchers also try to explore ITH from other perspectives than subclones. For example, Oesper et al^36^ developed an algorithm known as “tumor heterogeneity analysis” that can assess the level of CNV heterogeneity within a tumor.

In recent years, noncoding DNA has been used by researchers to track ITH. Naxerova et al^32^ proposed the construction of phylogenetic trees using polyguanine, a hypervariable region in DNA. This may be a more convenient way to determine the source of ITH compared with the use of coding DNA. Base insertions and deletions in the polyguanine region can be detected. A similar number of insertions and deletions in the same polyguanine tract may be used as markers specific to a subclone. The authors included 22 patients and found that the insertion and deletion within the polyguanine region could be detected in 91% of the tumors. After establishing phylogenetic trees, it was evident that the primary sites of multiple tumor metastases are composed of multiple subclones, and the metastatic tumor is related to a specific subclone within the primary tumor.^8^ In colorectal cancer, the polyguanine technique is robust for determining the relationship between lymphatic and distant metastasis. In a study of 17 patients, lymphatic and distant metastases arose from two independent subclones from the primary lesion in 11 patients. Of the remaining six patients, lymphatic and distant metastasis were homologous. This provides more evidence for a linear (Tumor cells move from the primary lesion to the lymph nodes and subsequently to the distant organs orderly) or parallel (Tumor cells independently metastasize to lymph nodes and distant organs) metastasis model in colorectal cancer. In fact, Naxerova et al^32^ suggested that different patients with colorectal cancer may have different metastasis patterns.

When studying ITH, multiregion sampling is usually adopted. With respect to tumor sampling, multiple angles should be considered to cover multiple tissues at different depths.^37^ Some studies have indicated that sampling at three tumor‐rich sites rich may reduce the negative results of DNA testing, increase the accuracy of gene detection and improve overall ITH assessment.^38^ Sequencing tumor tissue DNA by multiregion sampling, however, may also be challenging. The contrast sampling method of tumor center, tumor periphery, and normal tissue obtained by diagnostic puncture method cannot achieve the function of multipoint sampling to obtain the information of intratumor heterogeneity.^39^ In addition, it is very difficult to obtain a multipoint sample that meets the test requirements in vivo. Therefore, many researchers have proposed the use of imaging to detect ITH to solve the problem. A method called “cancer rainbow” can fluorescently barcode somatic mutations, enabling the visualization of the expansion and diffusion of oncogenes. This allows direct observation of ITH within tumor tissue.^40^ Different levels of *VEGFR‐2* expression can also be determined in tumor tissue by molecular MRI.^41^ 18F‐FDG (18F‐flurodeoxyglucose) PET can track ITH based on blood flow and the metabolic rate of tumors.^42^ In addition, in vitro culturing of cells from multiple tumor sites can also be used to assess ITH indirectly.^43^ Such methods enrich the means of detecting ITH and may also establish a foundation for the clinical application of ITH in the future.

## 4 UNDERSTANDING CRC FROM THE PERSPECTIVE OF IPTH AND IITH

According to results from Jones et al^44^ and Mao et al,^45^ at least 50% of CRC patients have more than one heterogeneous region within their tumor. It is unclear whether this is the result of genetic or epigenetic differences, although all of them exhibit IPTH. Researchers have found that subclones containing *KRAS* and *NRAS* mutations can exist at different sites in primary CRC tumors.^46^ It is also possible that private *APC*, *TP53*, and *ERBB4* mutations exist in subclones from different tumor regions.^47^ In tumors with mutations of *KRAS*, *NRAS*, *PIK3CA,* or *BRAF*, the proportion of VAF with different mutations in tumor cell samples of the same patient varies significantly, which may also reflect the ITH of tumor cells.^48^ With respect to cell lines cultured in vitro, homogeneity is not observed either. It has been reported that there are three subclones in HT29 cells and four in the SW480 cell line.^49^ The studies above suggest that a tumor is a population consisting of different genotypes. Epigenetically, the expression levels of micro‐RNAs, such as miR‐92a and miR‐375, are significantly different in different areas of the tumor.^50^ Outside of the nucleus, private mutations and a high level of ITH can be detected in mitochondrial DNA (mtDNA) in different regions of the tumor tissue.^51^ In addition, the immune component of the tumor microenvironment may also have an impact on ITH. For example, infiltration of CD8^+^ and PD1^+^ T cells was significantly different in regions within a tumor.^52^

One of the most important features of advanced CRC is metastasis. IITH can provide us with more biological information about primary and matched metastatic lesions. Some studies have shown that *KRAS, NRAS*, *BRAF*, and *PIK3CA* mutations are homogenous in primary tumors and matched primary and metastatic lesions.^53^, ^54^ Metastatic lesions inherit multiple subclones from the primary site and the level of ITH in these lesions is relatively small. VAF in these lesions was significantly higher compared with the primary site.^13^, ^55^ The discussion above indicates that metastatic lesions are the result of subclones from the primary site that traveled from their original location to implant at distant sites. Furthermore, subclones in lymphatic metastases consist of early, middle, and late stage tumor cells during tumor progression, indicating that subclones from the primary site metastasize to lymph nodes at different points in the timeline.^56^ However, different metastatic lesions may contain different subclones from the primary site. In a study of 17 primary and matched metastasis samples, 6 of 17 tumors maintained the same origin of lymphatic and distant metastasis, while 11 of 17 tumors had different ancestors.^32^ CRC patients presenting with multiple primary sites may have a unique type of IITH. Using somatic copy number variation (SCNV) analysis, we found that a different primary site originated from different ancestors and their clone composition was also unique.^57^

## 5 ITH MAY PROVIDE TUMORS WITH SPECIFIC ABILITIES THAT RESULT IN WORSE OUTCOMES

There appears to be a significant correlation between ITH and cancer prognosis. In colorectal cancer patients, ITH index values correlated significantly with clinical prognosis.^58^ A decrease in overall survival (OS) associated with increased ITH has been found in a variety of cancers.^59^ For example, shorter OS and progression‐free survival (PFS) were observed in ovarian cancer patients with high ITH.^60^ In breast cancer patients, elevated estrogen receptor (ER) and ITH was found to be twice as fatal compared with patients exhibiting low ER and ITH.^61^ Similar results have been observed in CRC. Patients with higher ITH have shorter PFS.^58^ In metastatic CRC, the 3‐year OS and PFS of patients with low ITH in metastatic lesions were 66% and 23%, respectively, while patients exhibiting high ITH had shorter OS (18%) and PFS (5%).^62^ When using a tool called mutant‐allele tumor heterogeneity (MATH) to assess ITH in CRC patients, we also found that MATH was an independent risk factor for males.^63^ High ITH was also associated with a higher incidence of liver metastasis in CRC.^64^

In general, the biological characteristics of a tumor are determined by the dominant subclone population within, while a smaller proportion of subclones have little effect. The relationship between tumor subclones, however, is complex. Some subclones can promote proliferation of all the tumor cells by altering the tumor microenvironment.^65^ By implanting breast cancer cells with different genotypes from luminal and basal sites into wild‐type mice, researchers found the efficiency of tumorigenesis of a single type was very low. However, the mixed population at a 1:1 ratio could effectively induce tumor formation with clones comprised of genotypes from both sites.^66^ There seems to be a cooperative relationship between subclones in tumors. Each subclone acts like a gear in a machine that does its job. The population of a single subclone does not determine its overall importance. Rather, a small proportion of subclones within the tumor may play a vital role. Removal of these cells may lead to decreased tumor growth.^67^ The reason for this phenomenon may be that a specific subclone can change the immunogenicity of a tumor.^68^ In fact, it has been shown that there is a negative correlation between ITH and immune cell infiltration and tumors with higher ITH exhibit a lower level of immune T cell infiltration.^69^, ^70^

However, there are some controversies concerning how subclones affect cancer prognosis. Some researchers have found that compared to tumors with only one or two subclones, those with more than two subclones are associated with a poorer overall survival rate. However, no additional risk was observed in tumors with more than four subclones.^69^ Some studies have found that ITH has little effect on the growth rate of tumors when there are three or fewer subclones. But when the number of subclone increases to 6 or 12, the rate of tumor proliferation increases significantly.^70^ At present, the exact reason for the impact of ITH on prognosis is unclear. One possible explanation is that high ITH results in the interaction between subclones to induce a more complex tumor with a worse prognosis. It is also possible that high ITH results in an increased chance of obtaining more malignant subclones. Further study is needed in order to prove our hypotheses and observations.

## 6 ITH MAY CONFERS RESISTANCE TO TREATMENT BY PROVIDING SELECTABLE SUBCLONES OR COOPERATION BETWEEN SUBCLONES

**FIGURE 2:**
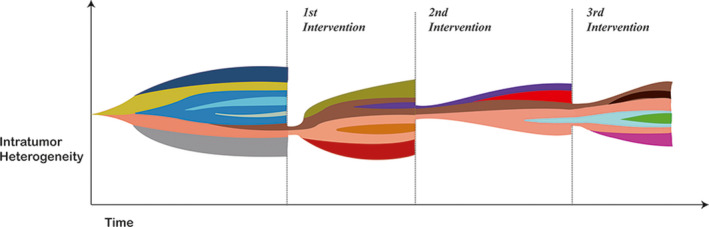
The level of intratumor heterogeneity during a series of interventions. A series of interventions serves as the selection pressure. There are no interventions in the beginning, thus subclones inside the tumor continue to increase due to neutral evolution. These subclones differ from each other genetically or epigenetically. The level of intratumor heterogeneity (the diversity of subclone population) gradually increases as time passes. After the first intervention, most subclones were wiped out while two subclones (colored in pink and brown), which have the ability to produce more offspring than others, continue to prosper and evolve into more genetically or epigenetically different subclones. After the second intervention, most subclones are eliminated while two (colored in pink and brown) remain to proliferate. The remaining subclones continue to evolve. The same process occurs during the third intervention. As the tumor is treated, the probability for the emergence of intervention‐resistant tumor cells gradually increases. (The color of the subclones gradually deepened.) It is also possible that intervention‐resistant tumor may obtain some biological characteristics that worsens the prognosis of the patients

ITH is closely linked to tumor resistance to treatment and tumors with high ITH exhibit greater drug resistance.^71^ When CRC patients receive neoadjuvant chemoradiotherapy, increased ITH results in the failure of follow‐up treatment.^72^ Various primary treatments may exert a strong selective effect and subclones may respond differently to treatment according to their genotype.^73^ Tumor cells that are susceptible to treatment will be eliminated as they cannot produce more offspring.^53^ Groups of resistant cells will then gain more survival advantages after intervention.^74^ Successive interventions may result in multiple selection of subclones with the highest level of resistance. As selections continue, the resistance to treatment will become stronger, and finally, the tumor will be uncontrollable (Figure 2). In addition, the development of CRC is likely to be a process that takes more than 20 years. In contrast, tumor metastasis may occur in the early stage of the tumor and remain dormant until activated.^11^ The effect of treatment intervention can change the proportion of the subclone population and create more space for these cells to grow.^75^ After obtaining space, the incubated cells begin to proliferate. They can also evolve into new subclones which transform the original sensitive group into a resistant colony after treatment.^76^ This also provides an explanation for the emergence of novel subclones carrying KRAS mutations in liver metastases from wild‐type RAS CRC patients treated with chemotherapy.^7^

Apart from providing more resistant subclones by heterogeneous tumors in the face of selection, the interaction between subclones will also lead to stronger drug resistance. *RAS^wt^* CRC is usually sensitive to cetuximab, while *KRAS^mut^* often results in drug resistance. The drug‐resistant phenotype of the *KRAS^mut^* subclone may not be limited to this specific subclone. Researchers found that TGF‐alpha and amphiregulin produced by tumor cells harboring a *KRAS* mutation can induce *KRAS^wt^* cells to grow continuously during drug exposure.^68^ PFS is similar in patients irrespective of the frequency of *KRAS* mutation.^45^ In a CRC patient cohort receiving neoadjuvant therapy, mutations in *APC*, *TP53*, *ABCA13*, *MUC16,* or *THSD4* are always present in the drug‐resistant population.^77^ This phenomenon has been verified in renal cell carcinoma patients using primary and matched metastatic lesions grown in nude mice.^78^ This evidence suggests that subclones from heterogeneous tumor subclones can cooperate with each other, creating a more powerful tumor.

## 7 LIMITATIONS AND PROSPECTS

There are some limitations that exist in the field of tumor biology from the perspective of ITH. Intratumor heterogeneity has not always been observed in tumors. For example, in a study of 88 breast cancer patients in which cells were collected from different regions of the tumors, only a few samples exhibited detectable ITH and the level of ITH was not associated with overall survival.^79^ The exploration of ITH is often derived from the inference of samples. Therefore, the sampling quality and the ability to take estimates on samples as a whole represents a limitation. Genotypic changes caused by a single point mutation may not be the cause of ITH. ITH may originate from mesoscale gene (about 10bp scale) or CNVs.^28^, ^80^ Therefore, it is possible that regular detection methods may fail to gather key information regarding ITH. The inference of ITH largely depends on specific algorithms. Existing algorithms that assess ITH must be evaluated to improve accuracy. Finally, the reason for the ITH effect on tumors is largely unknown and further studies are needed to explore these hidden mechanisms. Filling the information gap between ITH and clinical practice will benefit more patients. Despite many obstacles, continued understanding of ITH will enable us to trace the evolutionary pathway during tumor progression. In the area of CRC, this will facilitate our interpretation of disease development and prognosis. It may be said that one may not be afraid of long roads ahead with ITH, but afraid of short ambition.

## CONFLICT OF INTEREST

The authors have no financial or other conflict of interest to disclose.

## AUTHOR CONTRIBUTIONS

Zicheng Zheng was involved in conceptualization and writing—original draft. Tao Yu was involved in conceptualization, visualization, and writing—review and editing. Xinyu Zhao, Xin Gao, and Yao Zhao were involved in data curation. Gang Liu was involved in conceptualization, project administration, supervision, and writing—review and editing.

## Data Availability

No additional data are contained in this review.
